# Supplementary material for: Emerging Invasive Fungal Infections in Critically Ill Patients: Incidence, Outcomes and Prognosis Factors, a Case-Control Study
Source: J Fungi (Basel). 2021 Apr 24;7(5):330. doi: 10.3390/jof7050330 (PMC8146331; doi:10.3390/jof7050330)
Supplement: Supplementary file 1 [file jof-07-00330-s001.zip › jof-1164835-supplementary.pdf]

Supplementary Materials

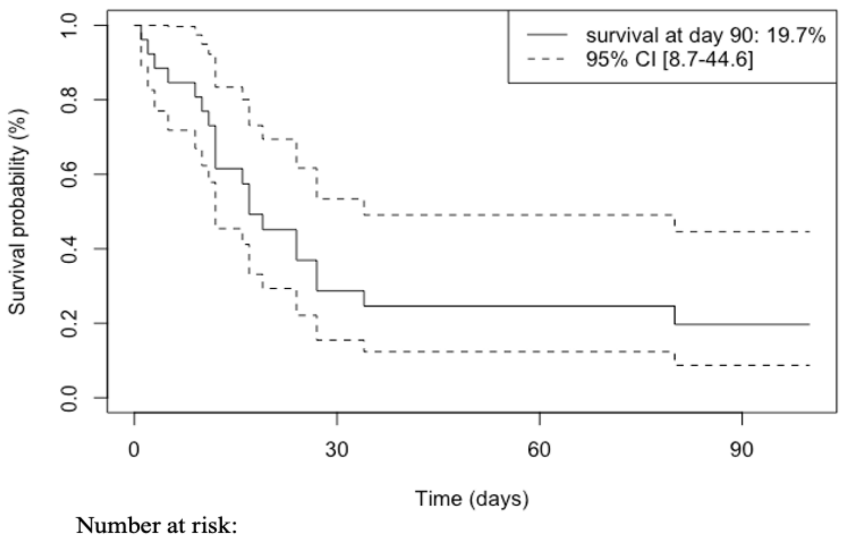

Number at risk:

n=26	n=7	n=6	n=3

Figure S1. Kaplan-Meier curve of day-90 survival after ICU admission of 26 patients with emerging invasive fungal infection (black line, the dashed lines represent the 95% confidence interval).

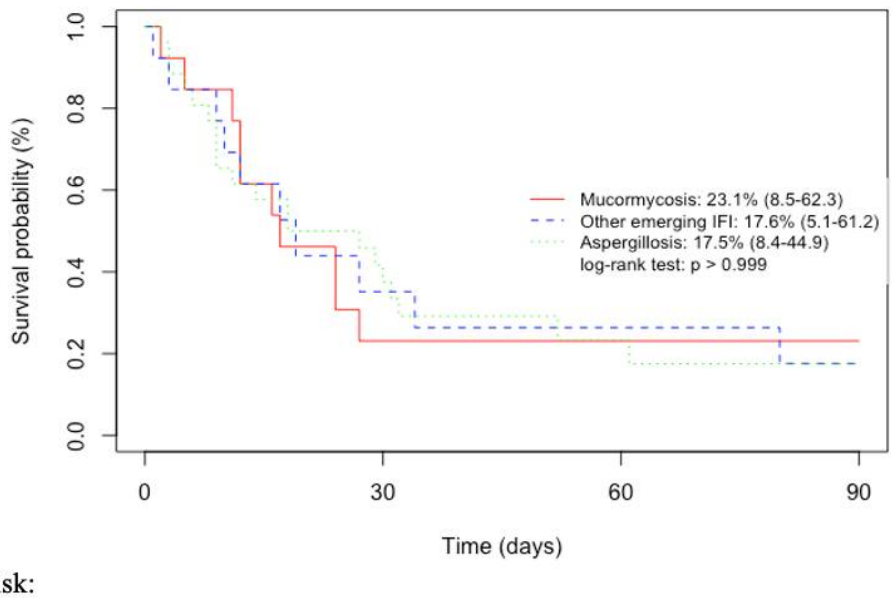

Number at risk:				
Aspergillosis:	n=26	n=10	n=4	n=3
Mucorales:	n=13	n=3	n=3	n=2
Other EIFIs:	n=13	n=4	n=3	n=1

Figure S2. Kaplan-Meier curves of day-90 survival after ICU admission for patients with aspergillosis (green dotted line), for those with mucormycosis (red line), and for those with other emerging invasive fungal infections (blue dashed line). Other emerging invasive fungal infections (EIFIs): *Saprochaete* (n=6), *Trichosporon* (n=1), *Fusarium* (n=2), *Scedosporium* (n=2), *Chaetomium* (n=1), *Saccharomyces* (n=1).

Table S1. Characteristics of the study population according to isolated mould.

	**Total (n=26)**	**Mucormycosis (n=13)**	**Other EIFI (n=13)**	**p-value**
Male, n (%)	21 (81%)	11 (85%)	10 (77%)	0.62
Age (years), median [IQR]	58.5 [47-69]	47 [41- 59]	68 [56-70]	0.01
Charlson index, median [IQR]	3 [2-5]	2 [2-3]	3 [3-5]	0.12
≥ 1 prior condition, n (%)	25 (96%)	12 (92%)	13 (100%)	0.99
Malnutrition, n (%)	21 (81%)	9 (69%)	12 (92%)	0.16
Candida colonization, n (%)	22 (85%)	11 (85%)	11 (85%)	0.92
Broad-spectrum antibiotic, n (%)	25 (96%)	12 (92%)	13 (100%)	>0.99
SAPS II, median [IQR]	59 [49-76]	61 [52-81]	58 [39-61]	0.35
SOFA at admission, median [IQR]	10 [8-13]	10 [8-13]	10 [8-11]	0.91
Invasive mechanical ventilation, n (%)	25 (96%)	13 (100%)	12 (92%)	>0.99
duration (days), median [IQR]	11 [4-24]	15 [4-24]	11 [10-24]	0.8
ARDS, n (%)	16 (62%)	7 (54%)	9 (69%)	0.42
Amines, n (%)	23 (89%)	11 (85%)	12 (92%)	0.55
duration (days), median [IQR]	13 [10-28]	13 [11-21]	13 [9-31]	0.45
AKI, n (%)	24 (92%)	12 (92%)	12 (92%)	>0.99
RRT, n (%)	18 (69%)	9 (69%)	9 (69%)	>0.99
duration, median [IQR]	10 [6-25]	13 [8-25]	10 [5-20]	0.31
Hepatic dysfunction, n (%)	18 (69%)	8 (62%)	10 (77%)	0.4
Myocardial dysfunction, n (%)	14 (54%)	5 (39%)	9 (69%)	0.12
Lymphocytes/mm^3, median [IQR]	155 [13-960]	340 [50-1000]	50 [10-650]	0.49
Pulmonary, n (%)	14 (54%)	6 (46%)	8 (62%)	0.43
Cutaneous, n (%)	9 (35%)	7 (53.8%)	2 (15%)	0.05
Blood, n (%)	8 (31%)	1 (7.7%)	7 (54%)	0.03
Sinus/orbit, n (%)	4 (15%)	4 (31%)	0 (0%)	>0.99
Cerebral, n (%)	2 (8%)	2 (15%)	0 (0%)	>0.99
Empiric antifungal therapy, n (%)	14 (54%)	7 (54%)	7 (54%)	>0.99
Antifungal therapy, n (%)	24 (92%)	12 (92%)	12 (92%)	>0.99
Adequate, n (%)	20 (77%)	10 (77%)	10 (77%)	>0.99
Amphotericin B, n (%)	18 (69%)	9 (69%)	9 (69%)	>0.99
Echinocandin, n (%)	6 (23%)	2 (15%)	4 (31%)	0.36
Azole, n (%)	5 (19%)	3 (23%)	2 (15%)	0.62
Flucytosine, n (%)	1 (4%)	0 (0%)	1 (8%)	0.99
Association, n (%)	5 (19%)	2 (15%)	3 (23%)	0.62
Surgery, n (%)	5 (19%)	5 (39%)	0 (0%)	>0.99
Post mortem EIFI diagnosis	10 (39%)	6 (46%)	4 (31%)	0.42
GCSF treatment, n (%)	8 (31%)	3 (23%)	5 (39%)	0.4
Immusuppress. therapy tapered, n (%)	11 (42%)	6 (46%)	5 (39%)	0.69
ICU LOS (days), median [IQR]	17 [9-27]	16 [11-24]	17 [9-34]	0.65
Deceased in ICU, n (%)	20 (77%)	10 (77%)	10 (77%)	>0.99
Deceased at day-90, n (%)	22 (85%)	11 (85%)	11 (85%)	>0.99

BMT: bone marrow transplantation (n=12), SOT: solid organ transplantation (kidney, n=2), Hematological mal.: hematological malignancies (n=14), COPD: chronic obstructive pulmonary disease, CKD: chronic kidney disease, CTD: connective tissue disease, LOS: length of stay, ICU: intensive care unit, SAPS II: Simplified Acute Physiology Score II, SOFA: Sequential Organ Failure Assessment, GCS: Glasgow coma scale, ARDS: acute respiratory distress syndrome, AKI: acute kidney injury (KDIGO stage ≥ 1), RRT: renal replacement therapy, EIFI: emerging invasive fungal infection, GCSF: Granulocyte Colony stimulating factor.
